# Dichlorido(3,5-dimethyl-1*H*-pyrazole-κ*N* ^2^)[hydro­tris­(3,5-dimethyl-1*H*-pyrazol-1-yl-κ*N* ^2^)borato]chromium(III) tetra­hydro­furan mono­solvate

**DOI:** 10.1107/S1600536811005344

**Published:** 2011-02-19

**Authors:** Li-Li Yuan, Chen Chen

**Affiliations:** a Department of Chemistry and Chemical Engineering, Hefei Normal University, Hefei, Anhui 230601, People’s Republic of China

## Abstract

In the title compound, [Cr(C_15_H_22_BN_6_)Cl_2_(C_5_H_8_N_2_)]·C_4_H_8_O, the Cr^III^ atom is coordinated by three N atoms from the hydro­tris­(3,5-dimethyl­pyrazol-1-yl)borate (Tp*) ligand, one 3,5-dimethyl­pyrazole (Dmpy) N atom and two Cl atoms in a distorted octa­hedral coordination geometry. Two N atoms occupy the axial sites, and the two Cl atoms and other two N atoms from Tp* lie in the equatorial plane. In the crystal, the complex mol­ecules and tetra­hydro­furan solvent mol­ecules are connected *via* inter­molecular N—H⋯O and C—H⋯O inter­actions.

## Related literature

For examples of the use of Tp*, see: Lobbia *et al.* (1991 ▶); Mashima *et al.* (1997 ▶); Nihei *et al.* (2010 ▶). For details of Cr(III) bonding, see: Wright-Garcia *et al.* (2003) ▶.
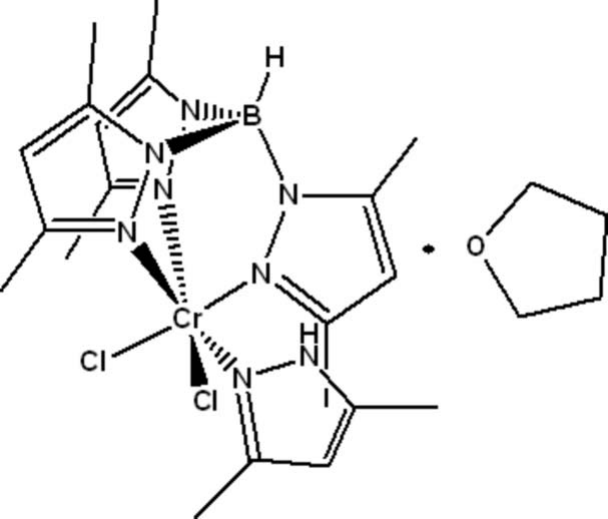


## Experimental

### 

#### Crystal data

[Cr(C_15_H_22_BN_6_)Cl_2_(C_5_H_8_N_2_)]·C_4_H_8_O
*M* *_r_* = 588.33
Monoclinic, 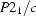

*a* = 10.9417 (13) Å
*b* = 11.1563 (13) Å
*c* = 24.036 (3) Å
β = 96.381 (2)°
*V* = 2915.9 (6) Å^3^
*Z* = 4
Mo *K*α radiation
μ = 0.61 mm^−1^
*T* = 298 K
0.2 × 0.2 × 0.2 mm

#### Data collection

Bruker SMART CCD area-detector diffractometer
Absorption correction: multi-scan (*SADABS*; Bruker, 2000 ▶) *T*_min_ = 0.886, *T*_max_ = 0.886
24008 measured reflections
5422 independent reflections
4930 reflections with *I* > 2σ(*I*)
*R*_int_ = 0.108

#### Refinement

*R*[*F* ^2^ > 2σ(*F* ^2^)] = 0.088
*wR*(*F* ^2^) = 0.189
*S* = 1.33
5422 reflections
351 parameters
H atoms treated by a mixture of independent and constrained refinement
Δρ_max_ = 0.64 e Å^−3^
Δρ_min_ = −0.36 e Å^−3^

### 

Data collection: *SMART* (Bruker, 2000 ▶); cell refinement: *SAINT* (Bruker, 2000 ▶); data reduction: *SAINT*; program(s) used to solve structure: *SHELXS97* (Sheldrick, 2008 ▶); program(s) used to refine structure: *SHELXL97* (Sheldrick, 2008 ▶); molecular graphics: *SHELXTL* (Sheldrick, 2008 ▶); software used to prepare material for publication: *SHELXTL*.

## Supplementary Material

Crystal structure: contains datablocks I, global. DOI: 10.1107/S1600536811005344/br2160sup1.cif

Structure factors: contains datablocks I. DOI: 10.1107/S1600536811005344/br2160Isup2.hkl

Additional supplementary materials:  crystallographic information; 3D view; checkCIF report

## Figures and Tables

**Table 1:** Hydrogen-bond geometry (Å, °)

*D*—H⋯*A*	*D*—H	H⋯*A*	*D*⋯*A*	*D*—H⋯*A*
C20—H20*C*⋯O1^i^	0.96	2.51	3.265 (8)	135
N8—H20⋯O1^i^	0.77 (4)	2.11 (4)	2.820 (6)	153.8

Symmetry code: (i) 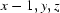.

**Fig. 1.**
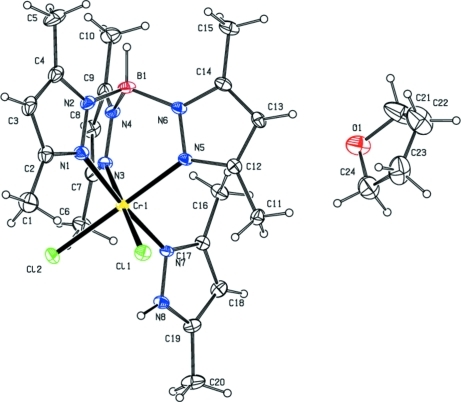
The molecular structure of the title complex, showing ellipsoids at the 30% probability level.

**Fig. 2.**
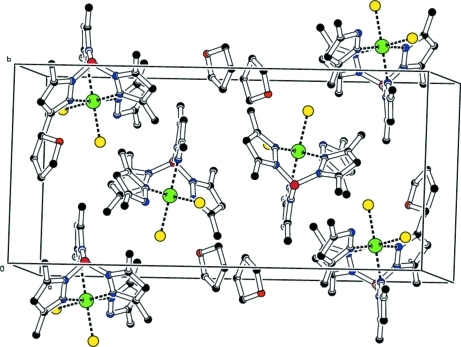
The molecular packing diagram of the title complex, with the hydrogen atoms ommitted.

## supplementary crystallographic information

### Comment

Since hydrotris(3,5-dimethylpyrazolyl)borate(Tp*) serves as tridentate, anionic, six-electron donor ligands, investigations of organometallic and inorganic chemistry using this type of ligand has developed rapidly (Mashima, *et al.*, 1997; Nihei, *et al.*, 2010; Wright-Garcia, *et al.*, 2003 and Lobbia, *et al.*, 1991). The molecular structure of title compound is shown in Fig.1. The Cr atom has an octahedral geometry and is coordinated by three N atoms from the tridentate Tp* ligand, one N atom from Dmpy ligand, and two Cl atoms. N3, N5 and Cl1, Cl2 lie in the equatorial plane. There are full non-classic hydrogen bonds in this complex. Intermolecules connect *via* hydrogen bond with C20 (Dmpy) and O1 (THF). A pair of THF uncoordination molecules around a center of symmetry exist in the stacking of copound.

The other coordinated bond angles are shown in Table 1. The molecular packing diagram of the title compound is shown in Fig.2.

### Experimental

Tp*SnCl_3_ was prepared according to the literature procedure (Mashima, *et al*.,1997). Treatment of Tp*SnCl_3_ (261 mg, 0.5 mmol) with CrCl_3_.3THF (187 mg 0.5 mmol) in 50 ml THF for 4 h at room temperature afforded a green solution that was evacuated to 20 ml, and the solution was carefully layered with 40 ml pentane. After 3 days at 253 K, green block crystals were obtained and were isolated *via* filtration.

### Refinement

The H atoms on some of the C atoms and on the B atom were located in a difference Fourier map and refined with the restraints C–H = 0.96–0.97) Å and B—H = 1.09 (5) Å,and N—H = 0.77 (4) Å, *U*_iso_(H) = 1.5Ueq(carrier). H atoms on pyrazolyl ring C atoms were placed in geometrically idealized positions and refined in riding mode, with C–H = 0.93 Å and *U*_iso_(H) = 1.2Ueq(C).

### Crystal data

**Table:**

[Cr(C_15_H_22_BN_6_)Cl_2_(C_5_H_8_N_2_)]·C_4_H_8_O	*Z* = 4
*M**_r_* = 588.33	*F*(000) = 1236
Monoclinic, *P*2_1_/*c*	*D*_x_ = 1.340 Mg m^−^^3^
Hall symbol: -P 2ybc	Mo *K*α radiation, λ = 0.71073 Å
*a* = 10.9417 (13) Å	θ = 1.0–25.5°
*b* = 11.1563 (13) Å	µ = 0.61 mm^−^^1^
*c* = 24.036 (3) Å	*T* = 298 K
β = 96.381 (2)°	Block, green
*V* = 2915.9 (6) Å^3^	0.2 × 0.2 × 0.2 mm

### Data collection

**Table:**

Bruker SMART CCD area-detector diffractometer	5422 independent reflections
Radiation source: fine-focus sealed tube	4930 reflections with *I* > 2σ(*I*)
graphite	*R*_int_ = 0.108
φ and ω scans	θ_max_ = 25.5°, θ_min_ = 1.7°
Absorption correction: multi-scan (*SADABS*; Bruker, 2000)	*h* = −11→13
*T*_min_ = 0.886, *T*_max_ = 0.886	*k* = −13→13
24008 measured reflections	*l* = −29→29

### Refinement

**Table:**

Refinement on *F*^2^	Primary atom site location: structure-invariant direct methods
Least-squares matrix: full	Secondary atom site location: difference Fourier map
*R*[*F*^2^ > 2σ(*F*^2^)] = 0.088	Hydrogen site location: inferred from neighbouring sites
*wR*(*F*^2^) = 0.189	H atoms treated by a mixture of independent and constrained refinement
*S* = 1.33	*w* = 1/[σ^2^(*F*_o_^2^) + (0.0478*P*)^2^ + 5.8124*P*] where *P* = (*F*_o_^2^ + 2*F*_c_^2^)/3
5422 reflections	(Δ/σ)_max_ = 0.061
351 parameters	Δρ_max_ = 0.64 e Å^−^^3^
0 restraints	Δρ_min_ = −0.36 e Å^−^^3^

### Special details

**Table:**

Geometry. All e.s.d.'s (except the e.s.d. in the dihedral angle between two l.s. planes) are estimated using the full covariance matrix. The cell e.s.d.'s are taken into account individually in the estimation of e.s.d.'s in distances, angles and torsion angles; correlations between e.s.d.'s in cell parameters are only used when they are defined by crystal symmetry. An approximate (isotropic) treatment of cell e.s.d.'s is used for estimating e.s.d.'s involving l.s. planes.
Refinement. Refinement of *F*^2^ against ALL reflections. The weighted *R*-factor *wR* and goodness of fit *S* are based on *F*^2^, conventional *R*-factors *R* are based on *F*, with *F* set to zero for negative *F*^2^. The threshold expression of *F*^2^ > σ(*F*^2^) is used only for calculating *R*-factors(gt) *etc*. and is not relevant to the choice of reflections for refinement. *R*-factors based on *F*^2^ are statistically about twice as large as those based on *F*, and *R*- factors based on ALL data will be even larger.

### Fractional atomic coordinates and isotropic or equivalent isotropic displacement parameters (Å^2^)

**Table:**

	*x*	*y*	*z*	*U*_iso_*/*U*_eq_	
H20	−0.049 (4)	0.229 (4)	0.8535 (17)	0.000 (14)*	
Cr1	0.21415 (6)	0.13372 (6)	0.86917 (3)	0.0257 (2)	
Cl1	0.21345 (12)	0.33718 (10)	0.84889 (5)	0.0403 (3)	
Cl2	0.12131 (12)	0.17258 (13)	0.94879 (5)	0.0443 (3)	
O1	0.8845 (5)	0.3794 (4)	0.8944 (2)	0.0768 (14)	
B1	0.4295 (5)	−0.0476 (5)	0.8590 (3)	0.0390 (13)	
N1	0.3892 (4)	0.1397 (4)	0.91225 (17)	0.0366 (9)	
N2	0.4684 (3)	0.0481 (4)	0.90276 (16)	0.0354 (9)	
N3	0.2138 (3)	−0.0490 (3)	0.88608 (16)	0.0324 (9)	
N4	0.3152 (4)	−0.1143 (3)	0.87563 (16)	0.0340 (9)	
N5	0.2998 (3)	0.0980 (3)	0.79742 (15)	0.0292 (8)	
N6	0.3927 (3)	0.0142 (3)	0.80210 (16)	0.0324 (9)	
N7	0.0439 (3)	0.1082 (3)	0.82229 (15)	0.0281 (8)	
N8	−0.0520 (4)	0.1813 (4)	0.83032 (19)	0.0327 (9)	
C1	0.3947 (6)	0.3210 (6)	0.9731 (3)	0.0663 (18)	
H1A	0.3673	0.3722	0.9421	0.099*	
H1B	0.4560	0.3618	0.9978	0.099*	
H1C	0.3263	0.3007	0.9930	0.099*	
C2	0.4486 (5)	0.2090 (5)	0.9518 (2)	0.0435 (13)	
C3	0.5638 (5)	0.1617 (6)	0.9677 (2)	0.0519 (15)	
H3	0.6229	0.1929	0.9946	0.062*	
C4	0.5747 (5)	0.0616 (6)	0.9367 (2)	0.0459 (14)	
C5	0.6814 (5)	−0.0219 (7)	0.9356 (3)	0.070 (2)	
H5A	0.6545	−0.1028	0.9404	0.105*	
H5B	0.7439	−0.0017	0.9654	0.105*	
H5C	0.7142	−0.0148	0.9004	0.105*	
C6	0.0046 (5)	−0.0972 (6)	0.9141 (3)	0.0526 (15)	
H6A	0.0070	−0.0197	0.9316	0.079*	
H6B	−0.0225	−0.1558	0.9393	0.079*	
H6C	−0.0513	−0.0953	0.8804	0.079*	
C7	0.1302 (5)	−0.1293 (5)	0.9003 (2)	0.0397 (12)	
C8	0.1793 (5)	−0.2434 (5)	0.8988 (2)	0.0451 (13)	
H8	0.1409	−0.3145	0.9071	0.054*	
C9	0.2944 (5)	−0.2318 (5)	0.8830 (2)	0.0410 (12)	
C10	0.3865 (6)	−0.3265 (5)	0.8738 (3)	0.0609 (17)	
H10A	0.4042	−0.3241	0.8356	0.091*	
H10B	0.3538	−0.4037	0.8817	0.091*	
H10C	0.4607	−0.3125	0.8982	0.091*	
C11	0.1907 (5)	0.2217 (5)	0.7202 (2)	0.0413 (12)	
H11A	0.1118	0.1834	0.7141	0.062*	
H11B	0.2128	0.2520	0.6853	0.062*	
H11C	0.1871	0.2867	0.7461	0.062*	
C12	0.2851 (4)	0.1326 (4)	0.7437 (2)	0.0333 (10)	
C13	0.3698 (5)	0.0739 (5)	0.7147 (2)	0.0396 (12)	
H13	0.3799	0.0830	0.6770	0.047*	
C14	0.4356 (4)	−0.0002 (4)	0.7523 (2)	0.0365 (11)	
C15	0.5383 (5)	−0.0845 (6)	0.7431 (3)	0.0543 (15)	
H15A	0.6119	−0.0601	0.7657	0.081*	
H15B	0.5520	−0.0831	0.7043	0.081*	
H15C	0.5167	−0.1643	0.7533	0.081*	
C16	0.0693 (5)	−0.0696 (5)	0.7607 (2)	0.0422 (12)	
H16A	0.1463	−0.0397	0.7508	0.063*	
H16B	0.0232	−0.1038	0.7283	0.063*	
H16C	0.0844	−0.1298	0.7892	0.063*	
C17	−0.0019 (4)	0.0306 (4)	0.78224 (19)	0.0315 (10)	
C18	−0.1248 (5)	0.0575 (5)	0.7659 (2)	0.0418 (12)	
H18	−0.1770	0.0179	0.7388	0.050*	
C19	−0.1540 (4)	0.1526 (4)	0.7972 (2)	0.0399 (12)	
C20	−0.2733 (5)	0.2176 (6)	0.7987 (3)	0.0641 (18)	
H20A	−0.3247	0.1736	0.8213	0.096*	
H20B	−0.3139	0.2251	0.7614	0.096*	
H20C	−0.2576	0.2960	0.8145	0.096*	
C21	0.8285 (10)	0.3483 (8)	0.9424 (5)	0.117 (4)	
H21A	0.8886	0.3138	0.9707	0.141*	
H21B	0.7636	0.2901	0.9331	0.141*	
C22	0.7779 (10)	0.4587 (9)	0.9632 (4)	0.114 (3)	
H22A	0.7904	0.4616	1.0038	0.137*	
H22B	0.6906	0.4648	0.9511	0.137*	
C23	0.8465 (10)	0.5566 (7)	0.9387 (4)	0.102 (3)	
H23A	0.8925	0.6037	0.9678	0.122*	
H23B	0.7910	0.6091	0.9158	0.122*	
C24	0.9310 (9)	0.4931 (7)	0.9039 (3)	0.088 (2)	
H24A	0.9346	0.5348	0.8687	0.105*	
H24B	1.0134	0.4894	0.9235	0.105*	
H1	0.504 (5)	−0.109 (4)	0.857 (2)	0.037 (13)*	

### Atomic displacement parameters (Å^2^)

**Table:**

	*U*^11^	*U*^22^	*U*^33^	*U*^12^	*U*^13^	*U*^23^
Cr1	0.0216 (4)	0.0282 (4)	0.0277 (4)	0.0017 (3)	0.0047 (3)	0.0002 (3)
Cl1	0.0453 (7)	0.0290 (6)	0.0468 (7)	−0.0007 (5)	0.0061 (5)	0.0009 (5)
Cl2	0.0448 (7)	0.0573 (8)	0.0325 (6)	0.0046 (6)	0.0124 (5)	−0.0039 (6)
O1	0.080 (3)	0.053 (3)	0.103 (4)	−0.007 (2)	0.038 (3)	−0.026 (3)
B1	0.024 (3)	0.039 (3)	0.055 (4)	0.008 (2)	0.009 (2)	0.009 (3)
N1	0.028 (2)	0.042 (2)	0.039 (2)	0.0012 (18)	0.0032 (17)	−0.0029 (19)
N2	0.025 (2)	0.043 (2)	0.037 (2)	0.0004 (18)	0.0010 (16)	0.0071 (18)
N3	0.031 (2)	0.031 (2)	0.036 (2)	0.0056 (17)	0.0095 (17)	0.0056 (17)
N4	0.032 (2)	0.032 (2)	0.039 (2)	0.0058 (17)	0.0053 (17)	0.0065 (17)
N5	0.025 (2)	0.030 (2)	0.034 (2)	0.0016 (16)	0.0105 (16)	0.0019 (16)
N6	0.025 (2)	0.035 (2)	0.039 (2)	0.0022 (16)	0.0113 (16)	0.0010 (17)
N7	0.025 (2)	0.0279 (19)	0.0328 (19)	0.0055 (15)	0.0074 (15)	−0.0001 (16)
N8	0.033 (2)	0.026 (2)	0.040 (2)	0.0021 (17)	0.0058 (18)	−0.0068 (19)
C1	0.056 (4)	0.074 (4)	0.066 (4)	−0.007 (3)	−0.009 (3)	−0.029 (3)
C2	0.038 (3)	0.051 (3)	0.040 (3)	−0.009 (2)	−0.002 (2)	−0.002 (2)
C3	0.036 (3)	0.077 (4)	0.039 (3)	−0.012 (3)	−0.010 (2)	0.002 (3)
C4	0.028 (3)	0.067 (4)	0.042 (3)	−0.003 (3)	−0.001 (2)	0.013 (3)
C5	0.032 (3)	0.086 (5)	0.088 (5)	0.011 (3)	−0.012 (3)	0.017 (4)
C6	0.045 (3)	0.056 (4)	0.062 (4)	−0.008 (3)	0.027 (3)	0.006 (3)
C7	0.039 (3)	0.041 (3)	0.040 (3)	−0.004 (2)	0.009 (2)	0.007 (2)
C8	0.052 (3)	0.034 (3)	0.051 (3)	−0.008 (2)	0.011 (3)	0.014 (2)
C9	0.051 (3)	0.035 (3)	0.037 (3)	0.003 (2)	0.004 (2)	0.006 (2)
C10	0.074 (4)	0.037 (3)	0.073 (4)	0.017 (3)	0.010 (3)	0.005 (3)
C11	0.046 (3)	0.042 (3)	0.036 (3)	0.003 (2)	0.003 (2)	0.009 (2)
C12	0.030 (2)	0.033 (2)	0.038 (3)	−0.008 (2)	0.008 (2)	−0.001 (2)
C13	0.038 (3)	0.048 (3)	0.035 (3)	−0.004 (2)	0.017 (2)	0.002 (2)
C14	0.028 (2)	0.037 (3)	0.048 (3)	−0.005 (2)	0.016 (2)	−0.003 (2)
C15	0.041 (3)	0.058 (4)	0.068 (4)	0.008 (3)	0.024 (3)	−0.003 (3)
C16	0.038 (3)	0.039 (3)	0.051 (3)	−0.009 (2)	0.009 (2)	−0.015 (2)
C17	0.030 (2)	0.027 (2)	0.037 (2)	−0.0058 (19)	0.007 (2)	0.000 (2)
C18	0.035 (3)	0.041 (3)	0.047 (3)	−0.009 (2)	−0.005 (2)	−0.002 (2)
C19	0.028 (3)	0.037 (3)	0.053 (3)	0.001 (2)	0.000 (2)	0.003 (2)
C20	0.034 (3)	0.058 (4)	0.098 (5)	0.008 (3)	−0.001 (3)	−0.006 (4)
C21	0.121 (8)	0.070 (5)	0.180 (11)	0.000 (5)	0.100 (8)	0.005 (6)
C22	0.141 (9)	0.096 (7)	0.116 (7)	0.021 (6)	0.068 (7)	−0.018 (6)
C23	0.166 (9)	0.060 (5)	0.080 (5)	0.010 (6)	0.015 (6)	−0.018 (4)
C24	0.126 (7)	0.065 (5)	0.075 (5)	−0.014 (5)	0.026 (5)	−0.010 (4)

### Geometric parameters (Å, °)

**Table:**

Cr1—N1	2.075 (4)	C7—C8	1.384 (7)
Cr1—N3	2.078 (4)	C8—C9	1.362 (7)
Cr1—N7	2.086 (4)	C8—H8	0.9300
Cr1—N5	2.090 (4)	C9—C10	1.493 (7)
Cr1—Cl2	2.3054 (14)	C10—H10A	0.9600
Cr1—Cl1	2.3214 (14)	C10—H10B	0.9600
O1—C24	1.376 (9)	C10—H10C	0.9600
O1—C21	1.410 (10)	C11—C12	1.498 (7)
B1—N2	1.527 (8)	C11—H11A	0.9600
B1—N6	1.544 (7)	C11—H11B	0.9600
B1—N4	1.545 (7)	C11—H11C	0.9600
B1—H1	1.07 (5)	C12—C13	1.385 (7)
N1—C2	1.336 (6)	C13—C14	1.370 (7)
N1—N2	1.375 (6)	C13—H13	0.9300
N2—C4	1.353 (6)	C14—C15	1.501 (7)
N3—C7	1.351 (6)	C15—H15A	0.9600
N3—N4	1.374 (5)	C15—H15B	0.9600
N4—C9	1.346 (6)	C15—H15C	0.9600
N5—C12	1.340 (6)	C16—C17	1.488 (7)
N5—N6	1.376 (5)	C16—H16A	0.9600
N6—C14	1.343 (6)	C16—H16B	0.9600
N7—C17	1.349 (6)	C16—H16C	0.9600
N7—N8	1.360 (5)	C17—C18	1.390 (7)
N8—C19	1.337 (6)	C18—C19	1.359 (7)
N8—H20	0.77 (4)	C18—H18	0.9300
C1—C2	1.496 (8)	C19—C20	1.497 (7)
C1—H1A	0.9600	C20—H20A	0.9600
C1—H1B	0.9600	C20—H20B	0.9600
C1—H1C	0.9600	C20—H20C	0.9600
C2—C3	1.381 (8)	C21—C22	1.462 (11)
C3—C4	1.355 (9)	C21—H21A	0.9700
C3—H3	0.9300	C21—H21B	0.9700
C4—C5	1.496 (8)	C22—C23	1.484 (13)
C5—H5A	0.9600	C22—H22A	0.9700
C5—H5B	0.9600	C22—H22B	0.9700
C5—H5C	0.9600	C23—C24	1.493 (11)
C6—C7	1.493 (7)	C23—H23A	0.9700
C6—H6A	0.9600	C23—H23B	0.9700
C6—H6B	0.9600	C24—H24A	0.9700
C6—H6C	0.9600	C24—H24B	0.9700
			
N1—Cr1—N3	87.45 (16)	C9—C8—H8	126.5
N1—Cr1—N7	173.26 (15)	C7—C8—H8	126.5
N3—Cr1—N7	87.18 (15)	N4—C9—C8	107.7 (5)
N1—Cr1—N5	86.80 (15)	N4—C9—C10	122.9 (5)
N3—Cr1—N5	89.14 (15)	C8—C9—C10	129.4 (5)
N7—Cr1—N5	89.05 (14)	C9—C10—H10A	109.5
N1—Cr1—Cl2	92.71 (12)	C9—C10—H10B	109.5
N3—Cr1—Cl2	90.66 (11)	H10A—C10—H10B	109.5
N7—Cr1—Cl2	91.42 (11)	C9—C10—H10C	109.5
N5—Cr1—Cl2	179.48 (11)	H10A—C10—H10C	109.5
N1—Cr1—Cl1	93.14 (12)	H10B—C10—H10C	109.5
N3—Cr1—Cl1	179.14 (12)	C12—C11—H11A	109.5
N7—Cr1—Cl1	92.19 (11)	C12—C11—H11B	109.5
N5—Cr1—Cl1	90.26 (11)	H11A—C11—H11B	109.5
Cl2—Cr1—Cl1	89.94 (5)	C12—C11—H11C	109.5
C24—O1—C21	106.1 (6)	H11A—C11—H11C	109.5
N2—B1—N6	108.9 (4)	H11B—C11—H11C	109.5
N2—B1—N4	109.2 (4)	N5—C12—C13	109.5 (4)
N6—B1—N4	107.8 (4)	N5—C12—C11	123.9 (4)
N2—B1—H1	109 (3)	C13—C12—C11	126.6 (4)
N6—B1—H1	112 (3)	C14—C13—C12	106.5 (4)
N4—B1—H1	110 (3)	C14—C13—H13	126.7
C2—N1—N2	106.4 (4)	C12—C13—H13	126.7
C2—N1—Cr1	136.2 (4)	N6—C14—C13	107.8 (4)
N2—N1—Cr1	117.2 (3)	N6—C14—C15	123.3 (5)
C4—N2—N1	109.4 (4)	C13—C14—C15	128.9 (5)
C4—N2—B1	130.5 (5)	C14—C15—H15A	109.5
N1—N2—B1	120.1 (4)	C14—C15—H15B	109.5
C7—N3—N4	106.1 (4)	H15A—C15—H15B	109.5
C7—N3—Cr1	135.7 (3)	C14—C15—H15C	109.5
N4—N3—Cr1	117.7 (3)	H15A—C15—H15C	109.5
C9—N4—N3	109.9 (4)	H15B—C15—H15C	109.5
C9—N4—B1	131.0 (4)	C17—C16—H16A	109.5
N3—N4—B1	119.0 (4)	C17—C16—H16B	109.5
C12—N5—N6	106.4 (4)	H16A—C16—H16B	109.5
C12—N5—Cr1	136.3 (3)	C17—C16—H16C	109.5
N6—N5—Cr1	117.2 (3)	H16A—C16—H16C	109.5
C14—N6—N5	109.7 (4)	H16B—C16—H16C	109.5
C14—N6—B1	130.7 (4)	N7—C17—C18	109.5 (4)
N5—N6—B1	119.6 (4)	N7—C17—C16	124.2 (4)
C17—N7—N8	104.7 (4)	C18—C17—C16	126.3 (4)
C17—N7—Cr1	135.4 (3)	C19—C18—C17	107.0 (4)
N8—N7—Cr1	119.9 (3)	C19—C18—H18	126.5
C19—N8—N7	112.2 (4)	C17—C18—H18	126.5
C19—N8—H20	124 (3)	N8—C19—C18	106.5 (4)
N7—N8—H20	123 (3)	N8—C19—C20	122.8 (5)
C2—C1—H1A	109.5	C18—C19—C20	130.7 (5)
C2—C1—H1B	109.5	C19—C20—H20A	109.5
H1A—C1—H1B	109.5	C19—C20—H20B	109.5
C2—C1—H1C	109.5	H20A—C20—H20B	109.5
H1A—C1—H1C	109.5	C19—C20—H20C	109.5
H1B—C1—H1C	109.5	H20A—C20—H20C	109.5
N1—C2—C3	109.4 (5)	H20B—C20—H20C	109.5
N1—C2—C1	123.2 (5)	O1—C21—C22	106.7 (7)
C3—C2—C1	127.4 (5)	O1—C21—H21A	110.4
C4—C3—C2	107.3 (5)	C22—C21—H21A	110.4
C4—C3—H3	126.4	O1—C21—H21B	110.4
C2—C3—H3	126.4	C22—C21—H21B	110.4
N2—C4—C3	107.5 (5)	H21A—C21—H21B	108.6
N2—C4—C5	122.7 (6)	C21—C22—C23	104.9 (7)
C3—C4—C5	129.8 (5)	C21—C22—H22A	110.8
C4—C5—H5A	109.5	C23—C22—H22A	110.8
C4—C5—H5B	109.5	C21—C22—H22B	110.8
H5A—C5—H5B	109.5	C23—C22—H22B	110.8
C4—C5—H5C	109.5	H22A—C22—H22B	108.8
H5A—C5—H5C	109.5	C22—C23—C24	104.2 (7)
H5B—C5—H5C	109.5	C22—C23—H23A	110.9
C7—C6—H6A	109.5	C24—C23—H23A	110.9
C7—C6—H6B	109.5	C22—C23—H23B	110.9
H6A—C6—H6B	109.5	C24—C23—H23B	110.9
C7—C6—H6C	109.5	H23A—C23—H23B	108.9
H6A—C6—H6C	109.5	O1—C24—C23	106.8 (7)
H6B—C6—H6C	109.5	O1—C24—H24A	110.4
N3—C7—C8	109.1 (5)	C23—C24—H24A	110.4
N3—C7—C6	124.3 (5)	O1—C24—H24B	110.4
C8—C7—C6	126.6 (5)	C23—C24—H24B	110.4
C9—C8—C7	107.1 (5)	H24A—C24—H24B	108.6

### Hydrogen-bond geometry (Å, °)

**Table:**

*D*—H···*A*	*D*—H	H···*A*	*D*···*A*	*D*—H···*A*
C20—H20C···O1^i^	0.96	2.51	3.265 (8)	135
N8—H20···O1^i^	0.77 (4)	2.11 (4)	2.820 (6)	153.8

Symmetry codes: (i) *x*−1, *y*, *z*.
